# Impact of neoadjuvant pembrolizumab adherence on pathologic complete response in triple-negative breast cancer: a real-world analysis

**DOI:** 10.1093/oncolo/oyae064

**Published:** 2024-04-24

**Authors:** Alexis LeVee, Megan Wong, Sarah Flores, Nora Ruel, Heather McArthur, James Waisman, Joanne Mortimer

**Affiliations:** Department of Medical Oncology, City of Hope Comprehensive Cancer Center, Duarte, CA, United States; Department of Medical Oncology, City of Hope Comprehensive Cancer Center, Duarte, CA, United States; Department of Medical Oncology, City of Hope Comprehensive Cancer Center, Duarte, CA, United States; Department of Medical Oncology, City of Hope Comprehensive Cancer Center, Duarte, CA, United States; Division of Hematology and Oncology, UT Southwestern Medical Center, Dallas, TX, United States; Department of Medical Oncology, City of Hope Comprehensive Cancer Center, Duarte, CA, United States; Department of Medical Oncology, City of Hope Comprehensive Cancer Center, Duarte, CA, United States

**Keywords:** triple-negative breast cancer, immunotherapy, pembrolizumab, pathologic complete response, neoadjuvant chemotherapy

## Abstract

**Background:**

The addition of pembrolizumab (pembro) to neoadjuvant chemotherapy (NAC) is standard of care for the treatment of early triple-negative breast cancer (TNBC) after KEYNOTE-522 trial demonstrated improved pathologic complete response (pCR) rates with the combination. However, the optimal treatment strategy for TNBC remains uncertain as questions persist about which patients benefit from pembro and the best treatment schedule and regimen. We identified real-world clinical characteristics and treatment variables associated with response to NAC plus pembro.

**Methods:**

Patients with early TNBC treated with NAC plus pembro between February 2020 and September 2023 were identified. Univariate and multivariate analysis was performed using logistic regression to identify factors associated with pCR. Cox proportional hazard prediction models were used to identify predictors of invasive disease-free survival and overall survival in this cohort.

**Results:**

A pCR was achieved in 75 (63.6%) of 118 patients. Age at diagnosis (*P* = .04), Ki-67 (*P* = .004), duration from start of pembro to surgery (*P* = .006) and NAC to surgery (*P* = .01), number of cycles of pembro (*P* = .04) and NAC (*P* = .02), and completion of at least 8 cycles of pembro (*P* = .015) and NAC (*P* = .015) were each significantly associated with pCR in univariate analysis. In multivariate analysis, patients younger than 55 years at time of diagnosis (vs age > 55 years) and those completing at least 8 cycles of pembro remained predictive of pCR (OR’s 2.50, 2.49, *P* = .035 and .037, respectively).

**Conclusions:**

In this real-world analysis of patients with TNBC treated with NAC plus pembro, younger age and the completion of at least 8 cycles of pembrolizumab were associated with pCR.

## Teaser

To date, there is no established biomarker to identify which patients will benefit from the addition of pembrolizumab to neoadjuvant chemotherapy and questions remain on the optimal treatment regimen. This study sought to identify real-world clinical characteristics and treatment variables associated with response to this regimen.

**Implications for Practice**

In this real-world analysis of patients with triple-negative breast cancer treated with neoadjuvant chemotherapy (NAC) plus pembrolizumab, younger age and the completion of neoadjuvant pembrolizumab were associated with pathologic complete response. This study suggests that patients should receive neoadjuvant pembrolizumab as clinically appropriate to optimize treatment outcomes with adequate consideration of toxicities.

## Introduction

Triple-negative breast cancer (TNBC) constitutes 10%-15% of all breast cancer cases and is defined by lack of estrogen receptor (ER), progesterone receptor (PR), and human epidermal growth factor receptor 2 (HER2).^1^ Compared to other subtypes, TNBC has a more aggressive natural history with higher rates of recurrence and mortality. Neoadjuvant chemotherapy (NAC) is the preferred approach for primary tumors greater than 2 cm and lymph node positive to shrink tumors before surgery and guide adjuvant therapies. Pathologic complete response (pCR) from the breast and lymph nodes after NAC is generally associated with improved survival and a reduced risk of relapse for TNBC, prompting its use as a surrogate marker of long-term outcomes.^2^ Traditional chemotherapy with anthracyclines and taxanes is the cornerstone of treatment but is most effective when administered on a dose-dense schedule and with the addition of platinum agents.^3-5^

Recent advances in TNBC have included the addition of immune checkpoint inhibitors (ICIs) to chemotherapy due to the immunogenicity of TNBC. For example, TNBC exhibits stromal tumor-infiltrating lymphocytes, programmed cell-death ligand 1 (PD-L1) expression, and immunosuppressive gene activity.^6-8^ In the metastatic setting, the addition of pembrolizumab to chemotherapy improved progression-free survival and overall survival (OS) in patients with TNBC whose tumors expressed PD-L1 with a combined positive score of 10 or more.^9,10^. In stages II and III TNBC, the phase III KEYNOTE-522 trial demonstrated that the addition of pembrolizumab to placebo improved pCR rates from 51.2% to 64.8% (*P* < .001) and event-free survival (EFS) from 76.8% to 84.5% (*P* < .001), regardless of PDL1 status.^11,12^ However, other ICIs have shown mixed results.^13-15^ Based on the results from KEYNOTE-522, current guidelines recommend the addition of pembrolizumab to 4 cycles of carboplatin and paclitaxel (TC) followed by 4 cycles of an anthracycline and cyclophosphamide (AC).^16^

The optimal neoadjuvant treatment strategy for an individual patient with TNBC is still unanswered. Subanalyses of KEYNOTE-522 showed that patients benefit from the addition of pembrolizumab regardless of nodal status, tumor size, carboplatin schedule, PD-L1 status, age, and ECOG performance status.^11^ Outcomes based on pembrolizumab exposure were not addressed. Because the addition of pembrolizumab can have fatal immune-related adverse events, it is critical that we identify which patients benefit most from the addition of pembrolizumab to NAC and the amount of pembrolizumab needed to improve disease-specific outcomes. To date, there is no established biomarker to identify which patients will benefit from this regimen and further studies are needed to identify optimal dosing regimens. As a result, we sought to identify real-world clinical characteristics and treatment variables associated with response to NAC plus pembrolizumab.

## Methods

A total of 177 patients with TNBC treated with NAC and at least one cycle of neoadjuvant pembrolizumab between February 2020 and September 2023 were identified using a City of Hope Comprehensive Cancer Center database. Patients were excluded if they were currently receiving neoadjuvant treatment (*n* = 43), had not undergone definitive surgery (*n* = 3), presented with distant metastatic disease at diagnosis (*n* = 11), and were missing surgical pathology data (*n* = 2). A total of 118 patients were included for analysis. TNBC was defined by the immunohistochemical (IHC) stain of ER and PR expression < 10 % and lack of HER2 expression or gene amplification. pCR was defined as ypT0/Tis and ypN0. Medical records through September 8, 2023 were reviewed, at which point data abstraction was cut off. Patients were censored at the date of last follow-up.

Patient’s demographic information (age at diagnosis, race/ethnicity, sex, and menopausal status) collected through the electronic medical record and tumor characteristics (tumor and node stage, histologic subtype, grade, and Ki-67 expression) were obtained from pathology reports of preneoadjuvant treatment core biopsy. Treatment data (dates of neoadjuvant treatment initiation and end date, type of chemotherapy, and number of cycles) were collected from the electronic medical record. Pathologic information was assessed from pathology reports of surgical specimens.

Patient and clinical characteristics were compared using 2 sample tests (*t* test or Wilcoxon rank-sum test for continuous variables and chi-square test for categorical data) between 2 groups defined by residual disease status. Univariate and multivariate logistic regression was performed to identify factors associated with pCR. Invasive disease-free survival (IDFS) and overall survival were calculated from the time of surgery. An IDFS event was defined as time to invasive ipsilateral breast tumor recurrence, local/regional invasive recurrence, distant recurrence, death from breast cancer, death from non-breast cancer, or death from an unknown cause, whichever presented first. Survival probability distribution was calculated using the Kaplan-Meier method, and Cox proportional hazards prediction models were used to identify predictors of IDFS and OS. A threshold of .05 was used to assess the significance of all statistical analyses. All analyses were conducted in SAS9.4. This study was approved by the City of Hope Comprehensive Cancer Center Institutional Review Board and was exempt from participant consent.

## Results

### Patient characteristics and treatment information

A total of 118 patients with TNBC treated with NAC and pembrolizumab followed by surgery were included in the analysis. Clinicopathologic data by residual disease status are summarized in Table 1. Among the 118 patients, all were female except for one male. Median age at diagnosis was 55.5 years (interquartile range [IQR] 46.6-62.1) and median body mass index (BMI as measured by kg/m) was 29.4 (IQR 23.9-32.3). Self-reported racial/ethnic groups included 42 non-Hispanic White (35.6%), 41 Hispanic/Latino (34.7%), 18 Asian (15.3%), 13 Black or African American (11.0%), and 4 other/unknown (4.3%). Most patients had invasive ductal histology (90.7%), clinical T2 stage (64.4%), and tumor grade 3 (81.4%). Over half of patients had node-negative (51.7%) disease at diagnosis.

**Table 1.** Patient characteristics and clinicopathologic outcomes according to pCR status.

	Non-pCR (*n* = 43)	pCR (*n* = 75)	*P-*value	All patients (*n *= 118)
Gender			.2	
F	42 (97.7%)	75 (100.0%)		117 (99.2%)
M	1 (2.3%)	0 (0.0%)		1 (0.8%)
Age at diagnosis, median (years) (IQR)	56.9 (49.4-67.4)	52.3 (44.8-61.8)	.04	55.5 (46.6-62.1)
BMI, median (kg/m^2^) (IQR)	29.7 (23.9-32.3)	29.2 (23.8-32.3)	.5	29.4 (23.9-32.3)
BMI group			.4	
Non-obese	23 (53.5%)	40 (53.3%)		63 (53.4%)
Obese/Morbidly obese	20 (46.5%)	35 (46.7%)		55 (46.6%)
Race and ethnicity			.4	
Asian	10 (23.3%)	8 (10.7%)		18 (15.3%)
Hispanic/Latino	14 (32.6%)	27 (36.0%)		41 (34.7%)
Non-Hispanic White	12 (27.9%)	30 (40.0%)		42 (35.6%)
Black or African American	5 (11.6%)	8 (10.7%)		13 (11.0%)
Other/unknown	2 (4.7%)	2 (2.7%)		4 (3.4%)
Comorbidities				
Diabetes Mellitus	9 (20.9%)	8 (10.7%)	*.1*	17 (14.4%)
Hypertension	11 (25.6%)	20 (26.7%)	*.9*	31 (26.3%)
Hyperlipidemia	16 (37.2%)	27 (36.0%)	*.9*	43 (36.4%)
Germline BRCA1 mutation			.7	
No/Unk	40 (93.0%)	68 (90.7%)		108 (91.5%)
Yes	3 (7.0%)	7 (9.3%)		10 (8.5%)
Germline BRCA2 mutation			.9	
No/Unk	42 (97.7%)	73 (97.3%)		115 (97.5%)
Yes	1 (2.3%)	2 (2.7%)		3 (2.5%)
Primary tumor classification			.8	
0^a^	0 (0.0%)	1 (1.3%)		1 (0.8%)
1	7 (16.3%)	10 (13.3%)		17 (14.4%)
2	29 (67.4%)	47 (62.7%)		76 (64.4%)
3	6 (14.0%)	11 (14.7%)		17 (14.4%)
4	1 (2.3%)	4 (5.3%)		5 (4.2%)
X	0 (0.0%)	2 (2.7%)		2 (1.7%)
Regional node classification			.7	
0	21 (48.8%)	40 (53.3%)		61 (51.7%)
1	18 (41.9%)	31 (41.3%)		49 (41.5%)
2	2 (4.7%)	3 (4.0%)		5 (4.2%)
3	1 (2.3%)	1 (1.3%)		2 (1.7%)
X	1 (2.3%)	0 (0.0%)		1 (0.8%)
Histopathology				
Ductal	37 (86.0%)	70 (93.3%)	.4	107 (90.7%)
Lobular	1 (2.3%)	0 (0.0%)		1 (0.8%)
Unspecified	1 (2.3%)	2 (2.7%)		3 (2.5%)
Other	4 (9.3%)	3 (4.0%)		7 (5.9%)
Tumor grade			.2	
2	9 (20.9%)	8 (10.7%)		17 (14.4%)
3	33 (76.7%)	63 (84.0%)		96 (81.4%)
Unknown	1 (2.3%)	4 (5.3%)		5 (4.2%)
Ki-67%, median (IQR)	60.0 (35.0-70.0)	75.0 (60.0-80.0)	.004^b^	70.0 (40.0-80.0)
Estrogen receptor, %			.2	
< 1	40 (93.0%)	73 (97.3%)		113 (94.9%)
1%-10%	3 (7.0%)	2 (2.7%)		5 (4.2%)
Progesterone receptor, %			.5	
< 1	41 (95.3%)	73 (97.3%)		114 (96.7%)
1%-10%	2 (4.6%)	2 (2.7%)		4 (3.3%)
HER2 IHC			.7	
0	20 (46.5%)	37 (49.3%)		57 (48.3%)
1+	12 (27.9%)	25 (33.3%)		37 (31.4%)
2+	8 (18.6%)	8 (10.7%)		16 (13.6%)
Not done	3 (7.0%)	5 (6.7%)		8 (6.8%)
HER2 FISH ratio			.3	
< 1.2	13 (31.7%)	26 (34.7%)		39 (33.6%)
1.2-2.2	12 (29.3%)	13 (17.3%)		25 (21.6%)
Not performed	16 (39.0%)	36 (48.0%)		52 (44.8%)
Duration from start of pembrolizumab to surgery, median (months; IQR)	5.2 (3.9-6.4)	6.3 (5.5-6.9)	.006	6.1 (4.9-6.8)
Duration from end of pembrolizumab to surgery, median (months; IQR)	1.1 (0.5-1.7)	1.3 (0.8-1.8)	.3	1.2 (0.7-1.8)
Duration from start of NAC to surgery, median (months; IQR)	5.8 (4.8-6.4)	6.3 (5.9-6.9)	.01	6.2 (5.5-6.8)
Duration from end of NAC to surgery, median (months; IQR)	1.3 (1.1-1.7)	1.3 (1.1-1.6)	.3	1.3 (1.1-1.7)
Number of cycles of neoadjuvant pembrolizumab, median (IQR)	6.0 (4.0-8.0)	8.0 (6.0-8.0)	.04	7.0 (5.0-8.0)
Number of cycles of NAC (IQR)	8 (6-8)	8 (6-8)	.02	8 (7-8)
Patients who completed 8 or more cycles of neoadjuvant pembrolizumab	13 (30.2%)	40 (53.3%)	.015	53 (44.9%)
Patients who completed 8 cycles of NAC	22 (51.2%)	55 (73.3%)	.015	77 (65.3%)
Carboplatin received			.7	
< 75% of doses	21 (48.8%)	39 (52.0%)		60 (50.8%)
≥ 75% of doses	18 (41.9%)	32 (42.7%)		50 (42.4%)
Unknown	4 (0.3%)	4 (5.3%)		8 (6.8%)
Taxane received			.14	
< 75% of doses	8 (18.6%)	6 (8.0%)		14 (11.9%)
≥ 75% of doses	31 (72.1%)	65 (86.7%)		96 (81.4%)
Unknown	4 (9.3%)	4 (5.3%)		8 (6.8%)
Received >1 dose of anthracycline-cyclophosphamide			.1	
Unknown	2 (4.6%)	3 (4.0%)		5 (4.2%)
No	9 (20.9%)	6 (8.0%)		15 (12.7%)
Yes	32 (74.4%)	66 (88.0%)		98 (83.1%)
Median follow-up months (95%CI)	9.7 (7.0, 13.4)	9.5 (6.8, 11.3)	.3	9.7 (8.1, 11.2)
Recurred				
No	32 (74.4%)	74 (98.7%)		106 (89.8%)
Yes	11 (25.6%)	1 (1.3%)		12 (10.2%)
Local/regional	1	0		1
Metastatic	10	1		11
IDFS, median (months; 95%CI)	14.0 (12.3, NR)	NR (NR, NR)	<.0001	NR (NR, NR)
IDFS at 18 months (95%CI)	39.8 (15.0, 64.0)	96.7 (78.6, 99.5)		
OS, median (months; 95%CI)	22.7 (16.1, NR)	NR (NR, NR)	.0038	33.9 (22.7, NR)
OS at 18 months (95%CI)	79.1 (49.3, 92.5)	100% (NE, NE)		

^a^One patient was T0N2.

^b^Only 86/118 had available ki-67%.

Abbreviations: NAC, neoadjuvant chemotherapy; NE, Not estimable; NR, not reached.

Per the inclusion criteria, all patients received at least 1 cycle of NAC and at least one cycle of neoadjuvant pembrolizumab. 77 (65.3%) patients completed 8 cycles of NAC, whereas 53 (44.9%) patients completed 8 or more cycles of neoadjuvant pembrolizumab. Of the patients who did not complete 8 cycles of NAC and/or neoadjuvant pembrolizumab, most discontinued treatment due to toxicity. Other reasons for completing less than 8 cycles of NAC and/or pembrolizumab included different chemotherapy regimens and schedules, different pembrolizumab dosing, pembrolizumab added on later during neoadjuvant treatment, and concern for progression.

Older patients and the presence of hyperlipidemia were associated with early discontinuation of chemotherapy (Supplementary Table S1). There were no significant differences in patient characteristics in those who completed 8 or more cycles of pembrolizumab compared to those who did not (Supplementary Table S2). Most patients (51/53, 96.2%) who completed 8 or more cycles of pembrolizumab also completed 8 cycles of NAC (Supplementary Table S1). On the other hand, 51 of the 77 (66.2%) patients who completed 8 cycles of NAC also completed 8 cycles of pembrolizumab.

The median number of cycles of pembrolizumab was 7 (IQR 5-8), whereas the median number of cycles of NAC was 8 (IQR 7-8). The median time from start and end of neoadjuvant pembrolizumab to surgery was 6.1 months (IQR 4.9-6.8) and 1.2 months (IQR 0.7-1.8), respectively. The median time from start and end of neoadjuvant NAC to surgery was similar at 6.2 (IQR 5.5-6.8) and 1.3 months (IQR 1.1-1.7), respectively, which reflects their overlapping administration schedules.

### Patient characteristics and treatments according to pCR status

A total of 75 (63.6%) patients achieved pCR (Table 1). Demographic information and tumor characteristics for those with and without pCR were statistically significant by age at diagnosis (*P* = .04) and median Ki-67 (0.004), while other baseline characteristics such as BMI, race/ethnicity, and BRCA1/2 mutation status were comparable. Tumor characteristics including clinical tumor and node stage, histopathology, grade, ER %, PR %, and HER2 IHC score did not vary significantly between patients with and without pCR.

pCR was related to the completion of NAC and pembrolizumab. Of the patients who achieved pCR, 55 (73.3%) completed 8 or more cycles of NAC, whereas 22 (51.2%) patients with residual disease completed NAC (*P* = .015). Similarly, of the patients who achieved pCR, 40 (53.3%) completed 8 or more cycles of pembrolizumab, whereas 13 (30.2%) of those with residual disease completed 8 or more cycles of pembrolizumab (*P* = .015).

Although patients who achieved pCR and those with residual disease both completed a median of 8 (IQR 6-8) cycles of NAC, this was statistically significant (*P* = .02). Likewise, patients who achieved pCR completed a median of 8 (IQR 6-8) cycles of neoadjuvant pembrolizumab compared to a median of 6 (IQR 4-8) cycles of neoadjuvant pembrolizumab for those with residual disease (*P* = .04). The frequency distribution of number of cycles of NAC and neoadjuvant pembrolizumab according to pCR rate are depicted in Table 2.

**Table 2.** Cycles of neoadjuvant pembrolizumab and NAC according to pCR rate.

Neoadjuvant pembrolizumab cycles	*N*	pCR rate (95%CI)	Neoadjuvant chemotherapy cycles	*N*	pCR rate (95%CI)
1	5	40.0% (0, 100)	1	—	
2	6	33.3% (0, 88)	2	5	20.0% (0, 76)
3	4	50.0% (0, 100)	3	2	50.0% (0, 100)
4	9	55.6% (15, 96)	4	5	100%
5	10	60.0% (23, 97)	5	7	42.9% (0, 92)
6	11	63.6% (30, 98)	6	3	0.00%
7	14	57.1% (27, 87)	7	10	50.0% (12, 88)
8	35	85.7% (73, 98)	8	77	71.4% (61, 82)
≥ 9	18	55.6% (30, 81)	Unknown	9	55.6% (15, 96)
Unknown	6	50.0% (0, 100)			

The duration from start of NAC to surgery and the start of pembrolizumab to surgery overlapped considerably with the response explained by the completion of at least 8 or more cycles of NAC or pembrolizumab, respectively. Patients who achieved pCR had a longer interval from the start of pembrolizumab to surgery (*P* = .006) and start of NAC to surgery (*P* = .01), which reflects the completion of their neoadjuvant treatment.

In univariate logistic regression, the receipt of at least 8 cycles of pembrolizumab and the receipt of at least 8 cycles of NAC were associated with pCR (Table 3). This finding was synonymous with the durations from start of pembrolizumab and NAC to surgery, respectively, which were both associated with pCR. Patients who received at least one cycle of AC were also more likely to achieve pCR than those who received no cycles of AC (OR 3.09, *P* = .047).

**Table 3.** Univariate and multivariate logistic analysis to identify predictors of pCR.

	Univariate analysis		Multivariate analysis	
Factor	OR (95%CI)	*P* value	OR (95%CI)	*P* value
Age at diagnosis: < 55 years vs ≥ 55 years	1.7 (0.86, 3.74)	.15	2.50 (1.07, 5.86)	.035
*Race/Ethnicity*				
Hispanic vs non-Hispanic White	0.77 (0.30, 1.96)	.7		
Other vs non-Hispanic White	0.42 (0.16, 1.09)	.08		
BMI: ≥ 30 (obese/morb. obese) vs* < *30 (non-obese)	1.0 (0.47, 2.11)	1.0		
Grade: 3 vs 2	2.15 (0.76, 6.08)	.15		
Clinical tumor stage: cT3 or cT4 vs cT0, cT1, cT2	1.33 (0.49, 3.58)	.6		
Clinical nodal stage: N + vs N0	0.84 (0.39, 1.77)	.6		
ER %: < 1% vs ≥ 1%	0.36 (0.06,2.28)	.3		
PR %: < 1% vs ≥ 1%	0.56 (0.08, 4.14)	.6		
Mos. from start of pembrolizumab to surgery (cont.)	1.39 (1.08, 1.78)	.01		
Mos. from completion of pembrolizumab to surgery (cont.)	0.97 (0.77, 1.21)	.8		
Mos. from start of NAC to surgery (cont.)	1.47 (1.08, 2.00)	.013		
Mos. from completion of NAC to surgery (cont.)	0.79 (0.51, 1.23)	.3		
Completed 8+ cycles of neoadjvuant pembrolizumab: yes vs no	2.60 (1.16, 5.83)	.021	2.49 (1.06, 5.90)	.037
Completed 8 cycles of NAC: yes vs no	2.83 (1.21, 6.64)	.017		
Received > 1 dose of AC: yes vs no	3.09 (1.01, 9.44)	.047		
^*^Ki-67 (pre-surg), (cont.)	1.03 (1.01, 1.05)	.007		

^*^Thirty-three patients missing Ki-67 values; this variable was not entered into multivariate model.

Cycles of carboplatin and taxane were excluded from the multivariate model since they overlap significantly with variables that identify completion of pembrolizumab, neoadjuvant chemotherapy, and receipt of any anthracycline plus cyclophosphamide.

Abbreviations: AC, anthracycline plus cyclophosphamide; ER, estrogen receptor; NAC, neoadjuvant chemotherapy; PR, progesterone receptor; TC, taxane plus carboplatin.

Variables significant at <0.2 level in the univariate analysis were entered into the multivariate analysis and kept if overall significance threshold of 0.05 was met, except for Ki-67, which was incomplete for 33 patients. A total of 108 patients were used in multivariate analysis with non-missing data for relevant variables. Multivariate results showed that patients younger than 55 years at time of diagnosis (vs age ≥ 55 years) and those completing at least 8 cycles of pembrolizumab had improved rates of pCR (odds ratio [OR] 2.50, 2.49, *P* = .035 and .037, respectively).

### Prognostic impact of pCR

With a median follow-up of 9.7 months (range 8.1 to 11.2) after surgery, there were 12 IDFS events with 1 locoregional recurrence and 11 distant recurrences. All but one of the IDFS events occurred in patients with residual disease after neoadjuvant treatment. There were 6 deaths in the cohort, each occurring in patients with residual disease after neoadjuvant treatment. Median IDFS for patients who achieved pCR was not reached (NR) as compared to the median IDFS was 14.0 months (95% CI: 12.3, NR) for patients with residual disease after neoadjuvant treatment. Median OS was 22.7 months (95% CI: 16.1, NR) for patients with residual disease after neoadjuvant treatment as compared to NR for patients who achieved pCR.

**Figure 1.**
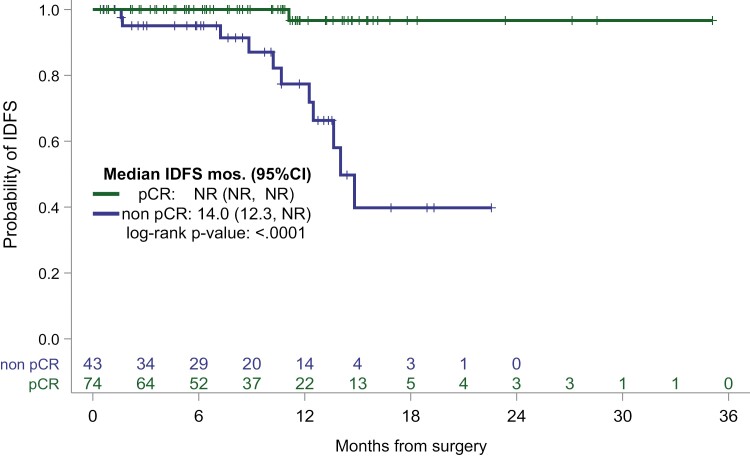
Invasive disease-free survival by pCR status.

**Figure 2.**
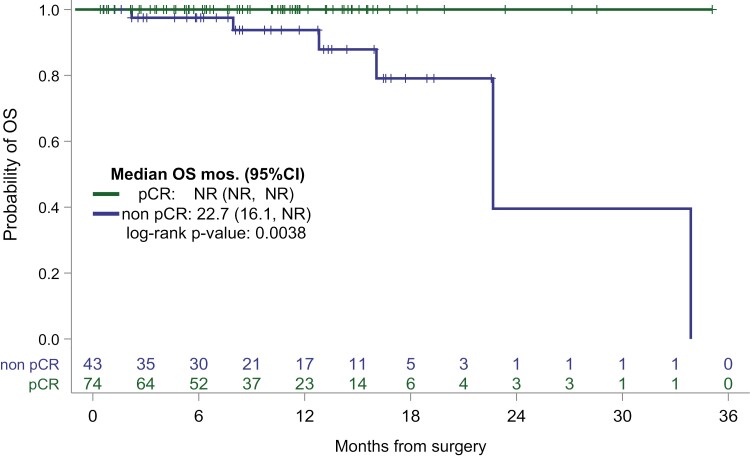
Overall survival by pCR status.

Kaplan-Meier curves for IDFS and OS according to pCR status after neoadjuvant treatment are demonstrated in Figures 1 and 2, respectively. Univariate Cox proportional hazards analysis was performed to identify predictors of IDFS in patients with residual disease after neoadjuvant treatment (Table 4). Analysis of IDFS was limited to patients with grade 3 tumors, as no IDFS events were observed in patients with grade 2. In this limited cohort of patients with grade 3 tumors and residual disease after neoadjuvant treatment (*n* = 33), node positivity was identified as the sole significant predictor of IDFS (hazard ratio [HR] 13.6, *P* = .015).

**Table 4.** Univariate Cox proportional hazards analysis to identify predictors of invasive disease-free survival in patients with residual disease after neoadjuvant treatment.

	IDFS univariate analysis	
Factor	HR (95%CI)	*P* value
Age at diagnosis: < 55 years vs ≥ 55 years	1.39 (0.42, 4.62)	.6
BMI: ≥ 30 (obese/morbidly obese) vs * < *30 (non-obese)	0.88 (0.26, 2.89)	.8
Grade: 3 vs 2		NE
Clinical tumor stage: cT3 or cT4 vs cT0, cT1, cT2	1.74 (0.35, 8.64)	.5
Clinical nodal stage: N + vs N0	2.80 (0.74, 10.6)	.13
Completed 8+ cycles of neoadjuvant pembrolizumab course (8 + cycles): yes vs no	1.86 (0.45, 7.63)	.4
Completed 8 cycles of NAC: yes vs no	2.21 (0.64, 7.84)	.2

Abbreviations: BMI, body mass index; IDFS, invasive disease-free survival; NAC, neoadjuvant chemotherapy; NE, not estimable Note: No IDFS events were in Grade 2 patients.

## Discussion

In this real-world analysis of patients with early-stage TNBC treated with NAC plus neoadjuvant pembrolizumab, the completion of at least 8 cycles of neoadjuvant pembrolizumab was associated with pCR. This suggests that patients should receive neoadjuvant pembrolizumab as clinically appropriate to increase their likelihood of achieving pCR. However, it is worth noting that the timelines for completing pembrolizumab and NAC overlapped considerably, potentially affecting these results.

In general, more cycles of both neoadjuvant pembrolizumab and NAC were associated with increasing likelihood of achieving pCR in subgroup analysis. However, due to overlapping treatment schedules and the small sample size of our cohort, these results remain exploratory. Several phase II and III clinical trials in early-stage TNBC evaluating the addition of neoadjuvant ICIs to NAC have used 8 cycles of ICI, whereas others have used less or more dictated by the NAC schedule.^13-15,17^ In the phase II ISPY-2 trial which investigated the addition of 4 cycles of neoadjuvant pembrolizumab to NAC, pCR rates more than doubled in the TNBC cohort.^17^ Given the observed benefit in the ISPY-2 trial with 4 cycles of neoadjuvant pembrolizumab coupled with the significant toxicity rates of the KEYNOTE-522 regimen, further clinical trials to establish the optimal dose and schedule of neoadjuvant pembrolizumab are needed.

Increasing age was also associated with a lower rate of pCR in patients treated with NAC plus pembrolizumab. This aligns with other prospective randomized controlled trials which show that younger patients are more likely to achieve pCR after NAC.^18^ Subgroup analyses from KEYNOTE-522 reaffirmed these findings, with patients aged less than 65 years demonstrating increased pCR rates (66.2%) as compared to patients equal to or above 65 years (54.3%).^11^ However, patients equal to or above 65 years still benefitted from the addition of pembrolizumab to NAC as the pCR rate of pembrolizumab plus NAC in these patients was higher as compared to that of the control arm (32%). Our study highlights the need for biomarkers to identify older patients who might not benefit from the addition of pembrolizumab to their neoadjuvant treatment regimen, sparing them the additional toxicity.

Examining diverse racial and ethnic groups revealed varying pCR rates, with White patients exhibiting the highest (71.4%) pCR rate, followed by Hispanic/Latino (65.9%), Black or African American (61.5%), and Asian patients (44.4%). Although these differences were not statistically significant due to small sample size, our study supports disparities in outcomes despite similar treatment regimens received as well as potential differences in tumor biology. In a recent prospective cohort study of patients treated with NAC, Black patients had lower rates of pCR after NAC compared to White patients and were more likely to have MAPK and PI3K/AKT pathway alterations.^19^ These alterations may be a potential mechanism of resistance underlying the inferior outcomes to treatment in Black patients. Further analysis is crucial to comprehend these differences fully and bridge existing gaps in outcomes among different racial and ethnic backgrounds.

Our study included patients with tumors of low ER and PR expression given that these patients are often treated as TNBC. Nevertheless, low (1%-10%) ER and PR expression are often excluded in clinical trials of TNBC due to the concern that this subgroup may behave differently as compared to ER less than 1%. Previous studies suggest that ER low-expressing tumors are biologically more similar to ER-negative tumors, classified predominantly as basal-like or HER2-enriched by PAM50 intrinsic subtyping.^20,21^ Low ER tumors have also been shown to behave more similarly to ER-negative tumors to NAC.^22,23^ In our study, tumors with low ER and PR expression responded similarly to ER- and PR-negative tumors, which suggests the benefit of adding pembrolizumab to NAC for this subgroup. The phase III KEYNOTE-756 trial which investigated the addition of pembrolizumab to NAC in early-stage high-risk ER-positive HER2-negative breast cancer reaffirmed these findings demonstrating improved pCR rates in patients whose tumors were ER < 10% as compared to ER > 10 %.^24^

With a median follow-up of 9.7 months (range 8.1 to 11.2) after surgery, there were 12 IDFS events in our cohort, with almost all occurring in patients with residual disease at the time of surgery. This supports the predictive power of pCR after NAC plus pembrolizumab in identifying individuals at reduced risk of relapse. Although the completion of at least 8 cycles of neoadjuvant pembrolizumab was not predictive of recurrence in patients with residual disease in univariate analysis, our analysis was limited by a small number of IDFS events. It also included data relating to neoadjuvant pembrolizumab exposure only and did not address adjuvant pembrolizumab. Ongoing trials such as the phase III OptimICE-pCR trial and SWOG S1418/NRG BR006 will help to answer whether adjuvant pembrolizumab improves long-term outcomes in patients with pCR and those with residual disease, respectively.

Updated results from KEYNOTE-522 after a median follow-up of 63.1 months demonstrate that neoadjuvant pembrolizumab plus NAC followed by adjuvant pembrolizumab improves EFS, regardless of pCR outcome.^25^ Other trials evaluating neoadjuvant ICIs in combination with NAC also show decreased disease recurrence despite differing pCR outcomes. For example, the GeparNuevo trial which evaluated the addition of neoadjuvant durvalumab to NAC showed improved IDFS despite a nonsignificant pCR increase,^15^ whereas the IMpassion031 trial which evaluated the addition of neoadjuvant atezolizumab to NAC showed both a pCR and EFS benefit.^26^ Although pCR is not a perfect surrogate of long-term clinical benefit with respect to the addition of neoadjuvant ICI, the addition of pembrolizumab in KEYNOTE-522 significantly improved 5-year EFS from 88.2% to 92.2% in those with a pCR compared to 52.3% to 62.6% in those without.^25^

This study is limited by small sample size which restricts our ability to draw meaningful conclusions relating to pembrolizumab dose intensity and pCR. In addition, chemotherapy dose reductions were not addressed, which can itself impact pCR. Finally, this study has a short follow-up time which limits our survival analyses. It does, however, provide a real-world experience. In addition, based on the real-world nature of the study, a control arm to compare pembrolizumab plus NAC could not be performed.

In conclusion, in this real-world analysis of patients with TNBC who received NAC plus pembrolizumab, younger patients and those who completed at least 8 cycles of neoadjuvant pembrolizumab were more likely to achieve pCR. These findings suggest that patients should receive neoadjuvant pembrolizumab as clinically appropriate to increase their chance of pCR. Further research is essential to refine the NAC and pembrolizumab regimen, ensuring the best treatment strategy for this high-risk population.

## Supplementary Material

oyae064_suppl_Supplementary_Table_S1

oyae064_suppl_Supplementary_Table_S2

## Data Availability

Data available on request from the authors.
