# Supplementary material for: Impact of neoadjuvant pembrolizumab adherence on pathologic complete response in triple-negative breast cancer: a real-world analysis
Source: Oncologist. 2024 Apr 24;29(7):566–74. doi: 10.1093/oncolo/oyae064 (PMC11224989; doi:10.1093/oncolo/oyae064)
Supplement: oyae064_suppl_Supplementary_Table_S1 [file oyae064_suppl_supplementary_table_s1.docx]

Table S1. Patient characteristics and clinical treatment according to the completion of chemotherapy.

|  | **8+ cycles (n=77)** | **<8 cycles (n=32)** | ***p*-value** |
| --- | --- | --- | --- |
| **Age at diagnosis, median (years) (IQR)** | 52.3 (45.5 - 59.6) | 59.2 (50.6 - 68.8) | 0.009 |
| **Gender** |  |  |  |
| F | 76 (98.7%) | 32 (100.0%) | 0.5 |
| M | 1 (1.3%) | 0 (0.0%) |  |
| **BMI, median (kg/m^2^) (IQR)** | 30.2 (24.1 - 32.3) | 27.7 (24.3 - 33.1) | 0.4 |
| **BMI group** |  |  |  |
| Not Obese | 38 (49.4%) | 19 (59.4%) | 0.3 |
| Obese/Morbidly obese | 39 (50.6%) | 13 (40.6%) |  |
| **Race and ethnicity** |  |  |  |
| Asian | 12 (15.6%) | 6 (18.8%) | 0.2 |
| Hispanic/Latino | 24 (31.2%) | 14 (43.8%) |  |
| Non-Hispanic White | 32 (41.6%) | 6 (18.8%) |  |
| Black or African American | 7 (9.1%) | 4 (12.5%) |  |
| Other/Unknown | 2 (2.6%) | 2 (6.3%) |  |
| **Comorbidities** |  |  |  |
| Diabetes Mellitus | 10 (13.0%) | 6 (18.8%) | 0.4 |
| Hypertension | 18 (23.4%) | 11 (34.4%) | 0.2 |
| Hyperlipidemia | 23 (29.9%) | 18 (56.2%) | 0.01 |
| **Primary tumor classification** |  |  |  |
| 0 | 1 (1.3%) | 0 (0.0%) | 0.8 |
| 1 | 13 (16.9%) | 4 (12.5%) |  |
| 2 | 49 (63.6%) | 20 (62.5%) |  |
| 3 | 11 (14.3%) | 6 (18.8%) |  |
| 4 | 2 (2.6%) | 2 (6.3%) |  |
| X | 1 (1.3%) | 0 (0.0%) |  |
| **Regional node classification** |  |  |  |
| 0 | 38 (49.4%) | 20 (62.5%) | 0.3 |
| 1 | 35 (45.5%) | 10 (31.3%) |  |
| 2 | 3 (3.9%) | 1 (3.1%) |  |
| 3 | 1 (1.3%) | 0 (0.0%) |  |
| X | 0 (0.0%) | 1 (3.1%) |  |
| **Histopathology** |  |  |  |
| Ductal | 72 (93.5%) | 27 (84.4%) | 0.3 |
| Lobular | 0 (0.0%) | 1 (3.1%) |  |
| Unspecified | 2 (2.6%) | 1 (3.1%) |  |
| Other | 3 (3.9%) | 3 (9.4%) |  |
| **pCR mean** (95% CL) | 71.4% (61.1, 81.7) | 46.9% (28.6, 65.2) | 0.015 |
| **Completed 8+ cycles of pembrolizumab** | 51 (66.2%) | 1 (3.1%) | <0.0001 |

Note: 9 patients with unknown number of chemotherapy cycles received were excluded from this table.
