# Supplementary material for: Impact of neoadjuvant pembrolizumab adherence on pathologic complete response in triple-negative breast cancer: a real-world analysis
Source: Oncologist. 2024 Apr 24;29(7):566–74. doi: 10.1093/oncolo/oyae064 (PMC11224989; doi:10.1093/oncolo/oyae064)
Supplement: oyae064_suppl_Supplementary_Table_S2 [file oyae064_suppl_supplementary_table_s2.docx]

Table S2. Patient characteristics and clinical treatment according to the completion of pembrolizumab.

|  | **8+ cycles (n=53)** | **<8 cycles (n=59)** | ***p*-value** |
| --- | --- | --- | --- |
| **Age at diagnosis, median (years) (IQR)** | 52.1 (45.5 - 60.8) | 56.4 (46.9 - 64.6) | 0.4 |
| **Gender** |  |  |  |
| F | 52 (98.1%) | 59 (100.0%) | 0.3 |
| M | 1 (1.9%) | 0 (0.0%) |  |
| **BMI, median (kg/m^2^) (IQR)** | 30.3 (25.3 - 33.6) | 27.1 (23.6 - 32.3) | 0.1 |
| **BMI group** |  |  |  |
| Not Obese | 24 (45.3%) | 35 (59.3%) | 0.1 |
| Obese/Morbidly obese | 29 (54.7%) | 24 (40.7%) |  |
| **Race and ethnicity** |  |  |  |
| Asian | 5 (9.4%) | 13 (22.0%) | 0.3 |
| Hispanic/Latino | 19 (35.8%) | 20 (33.9%) |  |
| Non-Hispanic White | 22 (41.5%) | 17 (28.8%) |  |
| Black or African American | 6 (11.3%) | 6 (10.2%) |  |
| Other/Unknown | 1 (1.9%) | 3 (5.1%) |  |
| **Comorbidities** |  |  |  |
| Diabetes Mellitus | 8 (15.1%) | 9 (15.2%) | 1.0 |
| Hypertension | 15 (28.3%) | 16 (27.1%) | 0.9 |
| Hyperlipidemia | 18 (34.0%) | 25 (42.4%) | 0.4 |
| **Primary tumor classification** |  |  |  |
| 0 | 1 (1.9%) | 0 (0.0%) | 0.6 |
| 1 | 9 (17.0%) | 8 (13.6%) |  |
| 2 | 34 (64.2%) | 38 (64.4%) |  |
| 3 | 6 (11.3%) | 11 (18.6%) |  |
| 4 | 2 (3.8%) | 2 (3.4%) |  |
| X | 1 (1.9%) | 0 (0.0%) |  |
| **Regional node classification** |  |  |  |
| 0 | 26 (49.1%) | 32 (54.2%) | 0.4 |
| 1 | 25 (47.2%) | 22 (37.3%) |  |
| 2 | 1 (1.9%) | 4 (6.8%) |  |
| 3 | 1 (1.9%) | 0 (0.0%) |  |
| X | 0 (0.0%) | 1 (1.7%) |  |
| **Histopathology** |  |  |  |
| Ductal | 48 (90.6%) | 54 (91.5%) | 0.7 |
| Lobular | 0 (0.0%) | 1 (1.7%) |  |
| Unspecified | 2 (3.8%) | 1 (1.7%) |  |
| Other | 3 (5.7%) | 3 (5.1%) |  |
| pCR mean (95%CL) | 75.4% (63.4, 87,4) | 54.2% (41.1, 67.3) | 0.02 |
| Completed 8 cycles of chemotherapy | 51 (96.2%) | 25 (42.4%) | <0.0001 |

Note: 6 patients with unknown number of pembrolizumab were excluded from this table.
